# System-level biological effects of extremely low-frequency electromagnetic fields: an *in vivo* experimental review

**DOI:** 10.3389/fnins.2023.1247021

**Published:** 2023-10-06

**Authors:** Haoyang Tian, Haozheng Zhu, Chenhao Gao, Mingxia Shi, Dekun Yang, Mingyu Jin, Fenghua Wang, Xiaohong Sui

**Affiliations:** ^1^Electric Power Research Institute, State Grid Shanghai Municipal Electric Power Company, Shanghai, China; ^2^School of Biomedical Engineering, Shanghai Jiao Tong University, Shanghai, China; ^3^State Grid Shanghai Municipal Electric Power Company, Shanghai, China; ^4^Department of Electrical Engineering, School of Electronic Information and Electrical Engineering, Shanghai Jiao Tong University, Shanghai, China

**Keywords:** ELF-EMF, health effect, electromagnetic radiation, power transmission line, work frequency

## Abstract

During the past decades, the potential effects of extremely low-frequency electromagnetic fields (ELF-EMFs) on human health have gained great interest all around the world. Though the International Commission on Non-Ionizing Radiation Protection recommended a 100 μT, and then a 200 μT magnetic field limit, the long-term effects of ELF-EMFs on organisms and systems need to be further investigated. It was reported that both electrotherapy and possible effects on human health could be induced under ELF-EM radiation with varied EM frequencies and fields. This present article intends to systematically review the *in vivo* experimental outcome and the corresponding mechanisms to shed some light on the safety considerations of ELF-EMFs. This will further advance the subsequent application of electrotherapy in human health.

## 1. Introduction

**Figure 1:**
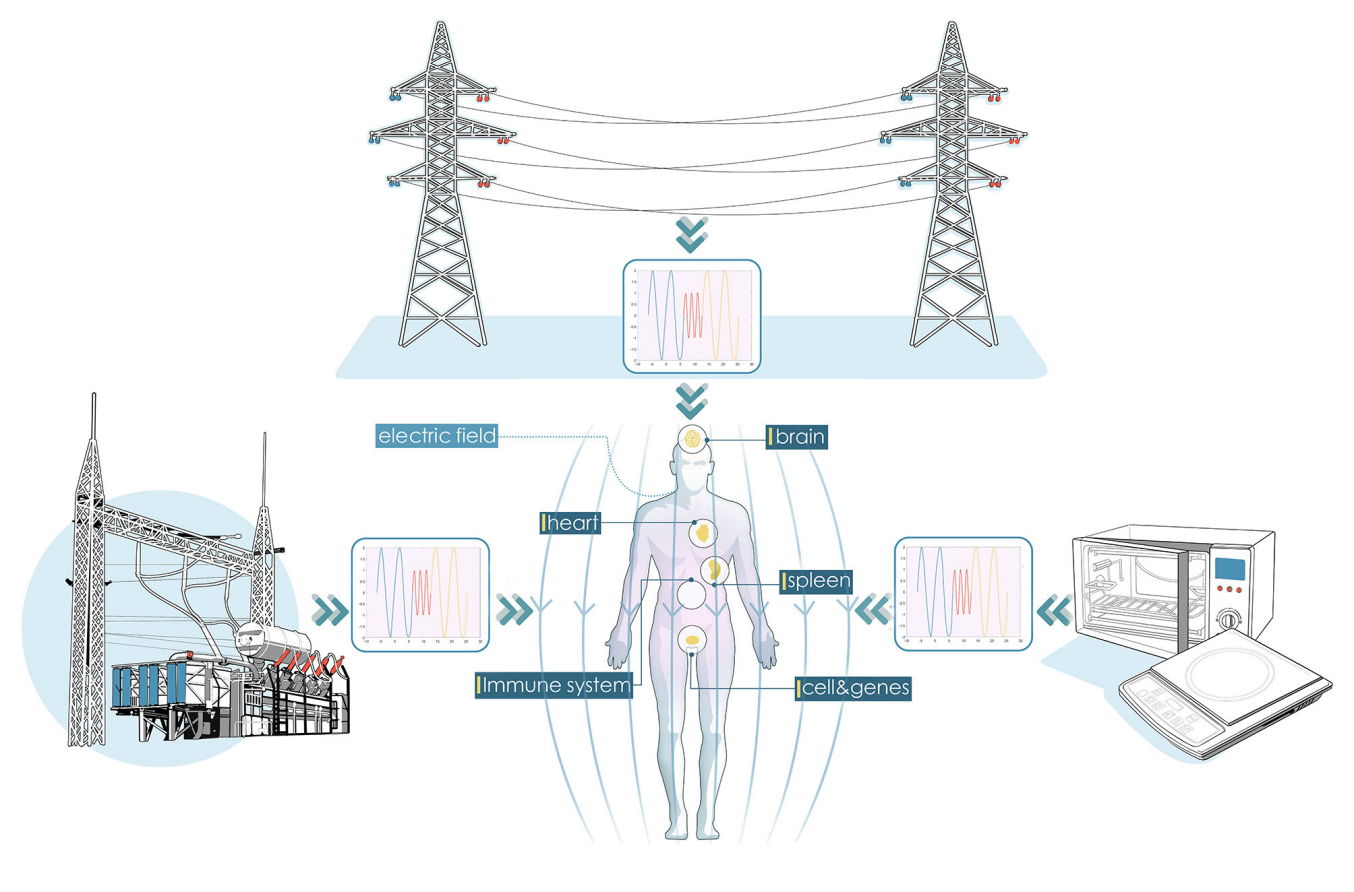
Illustration of biological effects of extremely low-frequency electromagnetic fields (ELF-EMFs) from power transmission lines, electrical appliances, etc.

Extremely low-frequency electromagnetic fields (ELF-EMFs) usually refer to alternating electromagnetic fields ranging from 1 to 100 Hz (Raggi et al., 2008) or 300 Hz (Sang-Kon et al., 2016), among which the power transmission frequency of 50 or 60 Hz is closely associated with our working and living environments. As shown in Figure 1, electrical fields can be produced by a high-voltage power transmission line, and induced current and magnetic fields would be generated inside biological systems, which might have a potential impact on the human body. During the past decades, the evaluation of the effects of ELF-EMFs on biological and human health has received significant attention (Carlberg et al., 2017; Turner et al., 2017; Consales et al., 2018; Jalilian et al., 2018; Zhang et al., 2018; Lee et al., 2020; Carnecka et al., 2022).

The International Commission on Non-Ionizing Radiation Protection (ICNIRP) recommended a magnetic induction limit of 100 μT for power frequency magnetic fields but further reassessed and revised the limitation to 200 μT in 2010 (ICNIRP, 2010) based on epidemiologic, *in vitro*, and *in vivo* studies of ELF-EMFs' health effects. Among all the research, experimental studies offer advantages over epidemiological ones, such as controllable variables, high repeatability, and the ability to support causal inferences. In the past few decades, experimental research on the biological effects of ELF-EMFs has made significant contributions at multiple levels, including genes, cells, tissues, and biological systems.

On the cellular level, biological activities include cell responses such as cell proliferation, differentiation, and apoptosis and resulting functional alterations, which can lead to either beneficial or adverse outcomes depending on the specific cell type and characteristics of the electromagnetic fields. Among them, cell proliferation is one of the most basic and crucial activities for cell growth, development, tissue repair, and structural and functional maintenance in organisms. On the one hand, some studies showed that exposure to ELF-EMFs had an inhibitory effect on cell proliferation. For instance, Manni et al. (2004) pointed out that human oral keratinocyte (HOK) cells exposed to an ELF-EMF of 2 mT at 50 Hz produced smaller clones than the control group and exhibited a decreased cell growth rate. In addition to normal cells, the findings from Lu et al. (2019) also confirmed this negative effect on tumor cell proliferation, possibly due to the induction of intracellular reactive oxygen species (ROS) under exposure to a combined static magnetic field and ELF-EMF of 5.1 mT at 50 Hz for 2h/day. Fathi and Farahzadi (2018) investigated the impact of ELF-EMF exposure on rat adipose tissue-derived mesenchymal stem cells (rADSCs) and observed a reduction in the proliferation rate of these cells. The same reduction was also validated by Cho et al. (2012) in human bone marrow-derived mesenchymal stem cells (hBM-MSCs) under a 12-day exposure to ELF-EMF. On the other hand, some other studies suggested that exposure to ELF-EMFs could promote cell proliferation, which further enhanced the therapeutic effects of low-frequency EMF on various diseases, including skin wounds (Manni et al., 2002; Huo et al., 2010; Lv et al., 2021) and orthopedic diseases (Li et al., 2018). Human keratinocytes (HaCaT) were found to have an increase in cellular growth and proliferation under ELF-EMFs through *in vitro* experiments (Manni et al., 2002; Cricenti et al., 2008; Vianale et al., 2008). The proliferation of rat calvarial osteoblasts and the formation of bone-like nodules were confirmed in Bodamyali et al. (1998) study under pulsed electromagnetic fields at 15 Hz for 6 h. The experiment conducted by Zhang et al. (2006) further manifested that a moderate-intensity magnetic field (1, 5, 10, and 20 mT) at 15 Hz with an exposure time of 30 min per day for 2 days further validated the significant increase of rat calvarial osteoblasts. *In vitro* experiments were further conducted on human epidermal stem cells, and the results supported the promotive effects of ELF-EMFs on cell proliferation in a frequency-dependent manner, with the most preferred frequency being 50 Hz (Zhang et al., 2013). In addition to cell proliferation, other cellular responses were also studied. An *in vitro* study by Kula et al. (2002) showed no obvious changes in the peroxidation of membrane structures in fibroblasts exposed to a static 0.49 T magnetic field or an ELF-EMF of 20 mT at 50 Hz. It was also reported that obese mice can lose weight under the treatment of 7.5 Hz, 0.4 T ELF-EMF, possibly due to the inhibition of adipogenic differentiation of mesenchymal stem cells, but there is no effect on osteogenic differentiation with ELF-EMF (Du et al., 2014).

The ELF-EMFs can affect the expression of some genes while exerting no obvious influence on others. For inhibitory effects, Mahaki et al. (2019a) showed that the expression levels of transcription factor Maf (c-Maf), signal transducer and activator of transcription 6 (STAT6), and retinoid-related orphan receptor alpha (RORa) in the spleen of rats decreased significantly after 2 months of exposure to the ELF-EMFs. Sun et al. (2009) showed that the frequency and exposure duration of the electromagnetic field had a great influence on the expression of the cell apoptosis-related proteins Bax and Bcl-2, and EMF would gradually reduce the expression of Bcl-2. Hu et al. (2006) showed that the adherence-related OPN gene expression level in vascular smooth muscle cells in ELF-EMF-exposed experimental groups was lower than that in the control group, and the inhibition effect was enhanced with the increase in EMF intensity. On the other hand, Ebrahimi et al. (2019) indicated that the expression levels of PROK and Cyp 17 increased, but the difference was not statistically significant. Mahaki et al. (2019a) showed that after 2 months of exposure to ELF-EMF, the expression of c-Maf, STAT6, and RORa in the thymus of rats did not decrease. Studies conducted by Reyes-Guerrero et al. (2010) indicated that there was no significant difference in the expression of estrogen receptor-α (ER-α) and estrogen receptor-β (ER-β) genes. Exposure to ELF-EMF during both prenatal and postnatal phases results in heightened IL-17A levels within the spleen and bloodstream of juvenile female rats. This exposure also triggered an increase in the expression of the IL-17 gene in the spleen, leading to the proliferation of CD4+ cells and an inflammatory response (Ozturk et al., 2022).

In the aforementioned studies on the cellular and gene level effects, although various studies have yielded either positive or negative findings, *in vitro* experiments alone cannot fully capture the interplay of diverse bodily systems in response to external stimuli. Therefore, *in vivo* studies are more suitable for comprehensively assessing the overall impact of experiments on living subjects. In this review, we aimed to systematically review *in vivo* experimental studies investigating the systemic effects of EMF and provide a comprehensive overview of the underlying mechanisms through selected *in vitro* experiments. In this way, we hope to address knowledge gaps and provide direction for future investigations.

## 2. Construction of extremely low-frequency electromagnetic field environment

In experimental research on the effects of ELF-EMFs, the accurate construction of controlled and reproducible environments is of paramount importance. The process of constructing appropriate ELF-EMF environments involves various considerations, including the choice of experimental apparatus, field strength, coil size, and turns per coil. Understanding the different methods and approaches used to generate ELF-EMF environments is essential for researchers seeking to design rigorous experiments and contribute to the advancement of knowledge in this field.

**Figure 2:**
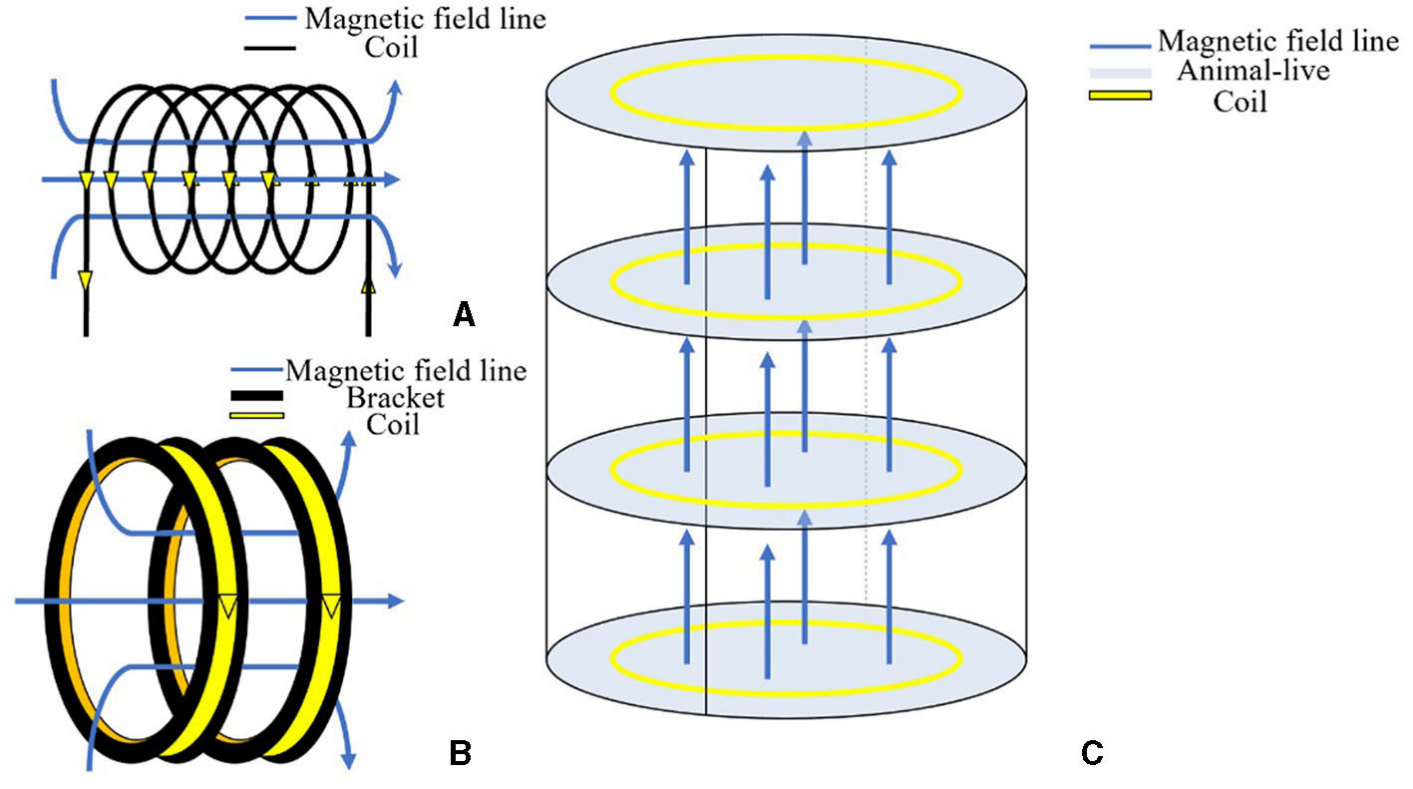
Construction of an extremely low-frequency electromagnetic field environment with a solenoid or coils. **(A)** Schematic diagram of generating a magnetic field with current passing through the solenoid based on the right-hand screw rule. **(B)** The reproduction of the magnetic field within the coils. **(C)** Schematic diagram illustrating the generation of a magnetic field in the animals' living space after the coils are energized.

Experimentally, there are two main methods to generate the ELF-EMF: either a single solenoid or coils parallel to each other, as shown in Figure 2. In the cell experiment of Zhang et al. (2006), the electromagnetic emission apparatus consisted of a tightly wound solenoid coil with an inner diameter of 5 cm and a length of 50 cm, ensuring a relatively uniform magnetic field along the axis near the center of the coil. Mahaki et al. (2019a) used solenoids with a diameter of 40 cm and a length of 2 m to explore the effects of ELF-EMFs on certain gene expression in the rat spleen and thymus. During the experiment, changeable field intensities of 1, 100, 500, and 2000 μT were generated by combining different turns of wire and voltage. However, the solenoid's excessive length of 2 m made it challenging to assess spatial homogeneity. The magnetic field generator built by Mao et al. (2018) was composed of a pair of copper coils with a vertical distance of 70 mm, and each coil had 160 turns with an inner diameter of 440 mm and a height of 70 mm. A voltage-regulating transformer was used to modulate the current added to the coil, and an AC voltage ammeter was utilized to detect the current in the coil. The results showed that the magnetic field was uniform and stable, with the frequency fixed at 50 Hz, and the magnetic induction intensity was continuously adjustable from 0 to 0.5 mT. As for the device constructed by Lu et al. (2019), the two 30 cm-diameter coils were placed 11 cm apart and could generate a static magnetic field and ELF-EMF at a frequency of 50 Hz by direct current. In addition to self-built copper coils, Helmholtz coils were also utilized by some groups (Griefahn et al., 2001; Jeong et al., 2005; Zhang et al., 2013) to generate ELF-EMF. In the experiment of Zhang et al. (2013), the two coils were 15 ± 0.3 cm in diameter and were placed 15 ± 0.5 cm apart. The results showed that ELF-EMF with a magnetic field intensity of 5 mT and a frequency of 1 to 50 Hz could be produced. To ensure sufficient living space for the animals in the experiment, large-diameter copper coils were employed in Zhang Y. et al.'s (2016) and Liu et al.'s (2018) studies. The device's size was 2 m in length, 1 m in width, and 2 m in height, with a distance between the adjacent coils of 0.35 m. Among the coils, the ones on the top and bottom were wrapped with 400 turns of copper wire, while the three coils in the middle were wrapped with 200 turns. After being connected to the voltage-regulating transformer and powered by the voltage-regulating power supply, a magnetic field with an intensity of 0–2 mT was generated.

In summary, *in vitro* experiments have lower spatial requirements and can achieve a uniformly distributed magnetic field using small-sized coils/solenoids. On the other hand, for animal experiments, where a uniform magnetic field is required in a larger space, coils are more preferred than solenoids.

## 3. Effects of ELF-EMFs on biological activities on the system level

### 3.1. Immune system

The impact of EMF on the immune system has garnered significant attention in recent years. The immune system comprises intricately structured cells, tissues, and organs. The functions of the thymus, spleen, and macrophage phagocytosis; serum levels of antibodies and cytokines; and the number of immune cells are common indicators for evaluating the immune system's function. Among them, the thymus, spleen, and macrophage phagocytosis indexes are closely associated with non-specific immune function, while the serum levels of antibody IgG can be measured in relation to humoral immune function. Existing reviews have summarized the cellular variations induced by EMF exposure as observed in epidemiological studies and *in vitro* experiments (Boscolo et al., 2007; Santini et al., 2009). However, to comprehensively evaluate the impact on the immune system's functionality, it remains crucial to consolidate conclusions derived from *in vivo* experiments. Table 1 shows that exposure to ELF-EMFs can have a positive or negative influence on biological immune function.

**Table 1:** ELF-EMF's effects on the immune system.

**References**	***In vitro*/*In vivo***	**Intervention description**	**Animal species**	**Main effect/Possible cause/ Mechanism**
Canseven et al. (2006)	*In vivo* and *in vitro* measurement of NK cytotoxic activity	50 Hz, 2 mT MF with a period of 4 h/day for 5 days	19 male guinea pigs	NK cell cytotoxic activity of the exposed animals was found decreased.
de Kleijn et al. (2016)	*In vivo*	ELF-EMF of 10 μT contained multiple frequencies (20–5000 Hz) for 1 week and 15 weeks, respectively	BalB/c mice	There was a significant increase in leukocyte counts under short-term exposure, but no differences were observed in long-term exposure. This increase may be caused by changes in the HPA stress axis. Their data suggested a role for stress regulation in observed leukocyte shifts.
Mahaki et al. (2020)	*In vivo*	1, 100, 500, and 2000 μT 50 Hz EMFs for 2 h/day for 60 days	80 adult male rats	Serum levels of IL-9 and TNF-α, as pro-inflammatory cytokines, were decreased due to 50 Hz EMF exposure compared with the controls in the pre- and post-stimulation phases.
Quaglino et al. (2004)	*In vivo*	50-Hz sinusoidal EMF for 8 months, two levels of field strength (1 kV/m, 5 mT; 5 kV/m, 100 mT)	2-month-old male Sprague-Dawley rats	Thymus from adult animals exhibits signs of gradual atrophy mainly due to collagen deposition and fat substitution.
Wyszkowska et al. (2018)	*In vivo*	A sinusoidal ELF-EMF (50 Hz, 7 mT) for either 1 h/day for 7 days, or continuously for 24 h	Male Wistar rats	A single continuous (lasting 24 h) exposure provoked a significant increase of the plasma IL-1β, IL-6, and IL-2 levels, and caused an elevation in blood parameters, such as white blood cells, lymphocytes, hemoglobin, and hematocrit levels. In contrast, repetitive exposure of rats to an ELF-EMF for 1 h/day for 7 days did not lead to any changes.
Zhang H. et al. (2016)	*In vivo*	ELF-EMF of 8 mT at 50 Hz	Male ICR mice	High-dose EMF decreases the concentration of thymus and spleen indexes and weakens the proliferation of T- and B-type lymphocytes.
Zhang Y. et al. (2016)	*In vivo*	100 μT magnetic field of 50 Hz for 20 h per day for 12 weeks	SD rats	The outcome showed no significant effects on the peripheral hematopoietic system without influencing the number of white blood cells, the neutrophil ratio, or the lymphocyte ratio.
Zhu (2010)	*In vivo*	1 mT, 4.5 mT, and 9 mT EMF for 8 months	BALB/c mice	The low-dose-treatment mice had increased thymus, spleen, and macrophage phagocytosis indexes, while high-dose EMF suppressed the immune function.

For immune organ function, Quaglino et al. (2004) conducted long-term *in vivo* exposure to 50-Hz ELF-EMF on Sprague-Dawley (SD) rats for 8 months, and the results demonstrated gradual atrophy in the rat thymus caused by collagen deposition and fat substitution. For studies in BALB/c mice, Zhu (2010) found that the low-dose-treatment mice induced an increase in thymus, spleen, and macrophage phagocytosis indexes at 1 mT and 4.5 mT. By comparison, all these indexes and levels of antibody IgG significantly decreased in the high-dose treatment group at 9 mT. This study indicated that low-dose ELF-EMFs were likely to contribute to human health, while a strong magnetic field could weaken the immune system. Zhang H. et al. (2016) denoted that an ELF-EMF of 8 mT at 50 Hz could decrease the concentration of thymus and spleen indexes and weaken the proliferation of T- and B-type lymphocytes, which implied that high-dose magnetic fields would have certain adverse effects on the biological immune system.

Immune cells such as white blood cells, lymphocytes, and natural killer (NK) cells play a vital role in the immune system. de Kleijn et al. (2016) exposed BalB/c mice to the ELF-EMF of 10 μT for 1 week and 15 weeks, respectively and observed a significant increase in leukocyte counts under short-term exposure. Furthermore, Zhang Y. et al. (2016) exerted a 100-μT magnetic field at 50 Hz on SD rats for 12 consecutive weeks, and the outcome showed no significant effects on the peripheral hematopoietic system without influencing the number of white blood cells, the neutrophil ratio, or the lymphocyte ratio. Canseven et al. (2006) applied a 50-Hz 2-mT EMF for 5 days to guinea pigs and found a decrease in NK cell cytotoxic activity in the spleens of the exposed animals.

Recently, researchers have shown increased interest in investigating alterations in cytokines in serum. Mahaki et al. (2020) exposed rats to 50 Hz EMFs at intensities of 1 and 100 μT and observed the reduction of pro-inflammatory cytokines such as IL-9 and TNF-α, alongside the increase of the anti-inflammatory cytokine IL-10, which could potentially trigger an anti-inflammatory response. The results from Wyszkowska et al. (2018) revealed that a single uninterrupted 24-h exposure to 50 Hz, 7 mT ELF-EMF resulted in notable elevations in plasma IL-1β, IL-6, and IL-2 levels, as well as an increase in blood parameters such as white blood cells, lymphocytes, hemoglobin, and hematocrit levels. Conversely, when rats were subjected to repetitive 1-h daily exposures to ELF-EMF over 7 days, there were no discernible alterations.

Based on the results from existing *in vitro* experiments, the mechanisms underlying the interaction between EMF and the immune system may involve calcium ion regulation, modulation of heat shock proteins, increased production of reactive oxygen species (ROS), cell signal transduction related to free radicals, and DNA damage and repair (Mahaki et al., 2019b). Certainly, some studies have investigated the immunomodulatory mechanisms of EMF and explored its potential therapeutic effects on certain diseases, such as wound healing (Rosado et al., 2018). However, on the whole, these mechanisms interplay, and impacts on the immune system can vary depending on factors such as exposure duration, frequency, and intensity, as well as the specific experiment subject types involved.

On the whole, in assessing immune organ function, *in vivo* experiments suggest that low-dose EMF exposure may be beneficial to human immune function, while high-dose exposure might have a weakening effect. The effects of EMF on immune cells and possible mechanisms depend on cell types and factors such as the intensity and duration of EMF exposure. The serum level of cytokines exhibited a possible anti-inflammatory effect to promote wound healing, but the impact depended on EMF parameters and might adapt to the changes with a longer exposure duration.

### 3.2. Reproduction

The effects of ELF-EMFs on reproduction can be quite concerning. In daily life, people pay special attention to the effect of electromagnetic radiation on fetal development. However, the adverse effects from epidemiological studies exhibited controversial results regarding both female and male reproductive functions as shown in Table 2. For instance, some studies showed no association between maternal or paternal exposure to ELF-EMFs and harmful reproductive outcomes either at 50 or 60 Hz (Sang-Kon et al., 2016). On the other hand, some cohort studies and case–control studies declared that women exposed to ELF-EMF during pregnancy might have a high risk of miscarriage (Shamsi Mahmoudabadi et al., 2013; Wang et al., 2013).

**Table 2:** ELF-EMF's effects on reproduction.

**Reference**	***In vitro/In vivo***	**Intervention description**	**Animal species**	**Main effect/Possible cause/ Mechanism**
Al-Akhras et al. (2001)	*In vivo*	A 50-Hz sinusoidal magnetic field of approximately 25 μT (rms) for 90 days	SD rats	The effects of the magnetic field on male fertility were negative but partly reversible.
Al-Akhras et al. (2006)	*In vivo*	A 50-Hz sinusoidal magnetic field at approximately 25 μT (rms) for 18 consecutive weeks	Male SD rats	There was a significant increase in the serum levels of male luteinizing hormone (LH) after 18 weeks of exposure (*P < * 0.005), while testosterone levels were significantly decreased only after 6 and 12 weeks of the exposure period.
Al-Akhras (2008)	*In vivo*	A 50-Hz sinusoidal magnetic field at approximately 25 microT (rms) for 18 weeks	Adult female Sprague-Dawley rats	Field exposure on SD rats showed a significant decrease in sex hormones including luteinizing hormone, progesterone, and estrogen, and a decrease in ovarian weight but the progesterone level was partly reversed after removing the exposure.
Aydin et al. (2009)	*In vivo*	Exposed continuously (24 h) to ELF-EMFs (48.21 +/– 1.58 mG) for 1, 2, and 3 months, respectively. The value of the EMF was calculated to be 0.48 +/– 0.05 mG	Adult Wistar female rats	The evaluation on hormonal change including progesterone level and 17-beta estradiol level showed no difference between the exposed group and control group.
Cao et al. (2006)	*In vivo*	21 days of exposure to the ELF-EMF of 1.2 mT at 50 Hz	Female Kunming mice	The weight increase of pregnant rats in the exposed group was significantly lower than that of the control group. The delivery rate of pregnant mice in the exposed group was significantly lower than that of the control group with the occurrence of preterm birth, stillbirth, and teratogenesis.
Heredia-Rojas et al. (2018)	*In vivo*	60 Hz and 8.8 μT EMFs during 72 h and 240 h	BALB/c mice	Low sperm counts were obtained for 72 h/2.0 mT-exposed animals (p < 0.05), without altering male germ cell morphological characteristics.
Ruan et al. (2020)	*In vivo*	30 μT, 100 μT, and 500 μT of magnetic field exposure for 20 h per day	SD rats and C57BL/6J mice	There was no significant difference in the mass or the number of stillbirths of pregnant rats.

*In vivo* experiments offer advantages over epidemiological studies in assessing the effects of EMF on the reproductive system due to controlled exposure and direct mechanistic insights (Karimi et al., 2020). Furthermore, *in vivo* experiments provide a method for exploring the effects of environmental factors on different compositions of the reproduction system in different periods of pregnancy (Asghari et al., 2016). Considering the weight effects of pregnant rats exposed to electromagnetic radiation, Cao et al. (2006) reported that the weight increase in pregnant mice in the exposed group was significantly lower than that of the control group in the late gestation period after 21 days of exposure to the ELF-EMF of 1.2 mT at 50 Hz. In addition, the delivery rate of pregnant mice in the exposed group was significantly lower than that of the control group, with the occurrence of preterm birth, stillbirth, and teratogenesis. Field exposure to SD rats showed a significant decrease in sex hormones, including luteinizing hormone, progesterone, and estrogen, and ovarian weight, but the progesterone level was partly reversed after removing the exposure (Al-Akhras, 2008). However, Ruan et al. (2020) found through animal experiments that there was no significant difference in the mass or the number of stillbirths among pregnant rats in each group after 30 μT, 100 μT, and 500 μT magnetic field exposure. The evaluation of hormonal changes, including progesterone level and 17-beta estradiol level, also showed no difference between the exposed group and the control group (Aydin et al., 2009). The mentioned studies confirmed the change in hormones due to exposure to EMFs, which might further influence the reproduction and fertility systems of animals, but the mechanism behind this needs to be explored. For male mice, ELF-EMF exposure led to lower sperm counts but did not alter germ cell morphological characteristics (Heredia-Rojas et al., 2018). Earlier rat models exhibited a reduction in reproductive outcomes after exposure to EMF for a short time but recovered in 90 days (Al-Akhras et al., 2001). Furthermore, a reduction in the level of serum testosterone was detected as a result of the reduced functionality of the seminal vesicles and preputial glands (Al-Akhras et al., 2006).

According to Santini et al. (2018) review of the effect of ELF-EMF on reproductive function, *in vivo* experiments since the early 2000s have exhibited complex results due to different experiment environments or animal species. For the mechanism, an increasing amount of research indicates that EMF exposure could lead to the overproduction of ROS by mitochondria in both male and female mice, which might explain its effect on the reproductive system (Santini et al., 2018), but further specific research is still required to delve into this matter due to the lack of clear mechanisms.

### 3.3. Cancer

In 2002, the International Agency for Research on Cancer (IARC) classified ELF-EMF as one of the possible carcinogenic factors for humans. In the past several decades, epidemiological studies have been conducted worldwide (Wertheimer and Leeper, 1979; Pedersen et al., 2014), but no concrete conclusion has been drawn regarding the relationship between ELF-EMF and various cancers (Carpenter, 2019). Therefore, to establish the causation between ELF-EMF and tumors, *in vivo* experiments were conducted to support the causal findings as shown in Table 3.

**Table 3:** ELF-EMF's effects on cancer.

**References**	***In vitro/In vivo***	**Intervention description**	**Animal species**	**Main effect/Possible cause/ Mechanism**
Barati et al. (2021)	*In vivo*	10-Hz 100-mT ELF-EMF for 2 h daily over a period of 28 days	BALB/c mice with MC-4L2 tumors	The research revealed a significant augmentation of proinflammatory reactions alongside the inhibition of tumor growth. The phenomenon was partly caused by ROS accumulation and Ca2+ overload-induced necroptosis.
Fedrowitz and Löscher (2007)	*In vivo*	A 100 μT, 50 Hz EMF for 26 weeks	Fischer rats with breast cancer	A significant increase in the incidence of adenocarcinomas was detected.
Galloni and Marino (2000)	*In vivo*	A 50 Hz, 2 mT EMF	Mammary murine adenocarcinoma-bearing mice	No association was observed.
McLean et al. (2003)	*In vivo*	A 60 Hz, 2 mT EMF for 29 weeks	SENCAR mice	No association was observed in the incidence of both benign and malignant skin tumors.
Salim et al. (2008)	*In vivo*	1 mT DC-MF (12 h/day)	SD rats with chemically induced colon cancer	The influence was neither carcinogenic nor cancer-promoting.
Soffritti and Giuliani (2019)	*In vivo*	Sinusoidal-50 Hz Magnetic Field	SD rats	No evidence showing the carcinogenic effects of ELF-EMF was detected.

*In vivo* experiments have shown mixed results. Galloni and Marino (2000) investigated the influence of a 50-Hz, 2 mT EMF on mammary murine adenocarcinoma-bearing mice, and no association was observed. For a 60-Hz, 2 mT EMF, the same results were observed in the incidence of both benign and malignant tumors (McLean et al., 2003). For rats, Salim et al. (2008) explored the effects of a 1 mT EMF on chemically induced colon cancer and revealed that the influence was neither carcinogenic nor cancer-promoting. The Ramazzini Institute had committed to filling the gap in long-term *in vivo* experiments with the use of more than 7000 SD rats, and no evidence showing the carcinogenic effects of ELF-EMF was detected (Soffritti and Giuliani, 2019). For breast cancer, Fedrowitz and Löscher (2007) exposed 344 Fischer rats to a 100 μT, 50 Hz EMF for 26 weeks and detected a significant increase in the incidence of adenocarcinomas. In conclusion, the results from *in vivo* tests can be controversial due to the animal species, cancer model types, and EMF parameters.

The carcinogenic mechanisms of ELF-EMF have been hypothesized to involve various factors, including the induction of DNA damage, cellular repair process impairment (Lai and Levitt, 2023), and altered hormone secretion. In acute experiments, Lai and Singh observed a dose-dependent increase in DNA strand breaks in brain cells in rats exposed to 60 Hz EMF, which could be related to carcinogenesis and cell death (Lai and Singh, 1997). However, in McNamee et al.'s (2002, 2005) experiments, adult rats, adult mice, and immature mice were exposed to 60 Hz EMFs, and no evidence supporting the hypothesis that the exposure might cause DNA damage in the cerebellum or brain was found. In long-term animal experiments, Yokus et al. (2005) measured the level of 8OHdG in DNA, a predominant form of radical-induced DNA lesions, and the results confirmed that an EMF of 0.97 mT at 50 Hz induced oxidative DNA damage. In conclusion, *in vivo* experiment results showed a difference in the effect of EMF on DNA damage. This could be due to the fact that the systems had diverse genetic characteristics (Santini et al., 2009), and *in vitro* experiments had already confirmed that the EMF effect was sensitive to cell types (Ivancsits et al., 2005). A hypothesis was put forward that the reduction of the hormone melatonin in the pineal gland might contribute to an increase in breast cancer (Loomis et al., 1994). To verify the hypothesis, Fedrowitz et al. (2002) utilized SD rats to observe the development and growth of mammary gland tumors and found that EMF increased the cell proliferation of mammary epithelium cells but did not influence melatonin levels.

Since some tumor cells go into apoptosis in response to ELF-EMFs (Yuan et al., 2020; Sołek et al., 2022), EMF has also been used for therapeutic purposes. This therapeutic effect on various cancers has long been demonstrated through *in vivo* experiments (De Seze et al., 2000; Tofani et al., 2002; Novikov et al., 2009; Mansourian et al., 2020; Chen et al., 2022). Several *in vitro* studies showed cell-type inhibition selectivity of EMF toward specific tumor cells other than normal cells (Koh et al., 2008; Crocetti et al., 2013), but the potential mechanism was still unknown, which necessitated further investigations to uncover the intricate workings at play. Recently, in a study by Barati et al. (2021), BALB/c mice with MC-4L2 tumors were exposed to a 10-Hz 100-mT ELF-EMF for 2 h daily over a period of 28 days. The research revealed a significant augmentation of proinflammatory reactions alongside the inhibition of tumor growth, and further verified through *in vitro* experiments that the phenomenon was partly caused by ROS accumulation and Ca^2+^ overload-induced necroptosis (Barati et al., 2021). In addition to the *in vivo* test, an *in vitro* experiment further proved that the inhibition mechanism was closely linked with the contact between cells and changes in membrane potential (Sun et al., 2023).

### 3.4. Cardiovascular system

The cardiovascular system is directly related to heartbeat and bioelectrical activities, which could potentially be affected by surrounding magnetic fields through inducing electric currents in biological body under the electromagnetic effects. A survey based on power company workers showed that there was a positive correlation between the duration of occupational electromagnetic field exposure and the incidence of arrhythmias and acute myocardial infarction (Savitz et al., 1999). On the whole, current epidemiological study findings identified limited evident cardiovascular risks associated with either short-term or prolonged exposures to ELF-EMFs within exposure limits set by current standards (Jauchem, 1997; McNamee et al., 2009).

Certain metrics, such as heart rate variability, heart rate, and blood pressure, are employed for the assessment and prediction of specific cardiovascular conditions. Since heartbeat and blood pressure are two key factors leading to cardiovascular disease, the effects of ELF-EMFs on heartbeat and blood pressure were mainly included in this review as summarized in Table 4. In short-term exposure, the results indicated that ELF-EMF might influence ventricular repolarization, leading to a suppression in heart rate elevation (Jeong et al., 2005). Other effects, including the reduction of glutathione content in the heart, were also detected in a 2-h exposure (Martínez-Sámano et al., 2010). For rat heart activity, no effect was observed under the exposure of a 50-Hz, 1-μT EMF (Elmas et al., 2012). To explore the mechanisms, Wei et al. (2015) exerted a 2 mT, 15 Hz, 50 Hz, 75 Hz, and 100 Hz EMF on cardiomyocytes isolated from neonatal SD rats and confirmed the modulation of calcium-related activities, including the increase in calcium concentration baseline level and the decrease in the amplitude of calcium transients in the sarcoplasmic reticulum. However, long-term experiments of 24 weeks on rats indicated that 50 Hz EMF had no obvious effects on the cardiovascular system through the detection of blood pressure, pulse rate, heart rate, and cardiac rhythm (Zhou et al., 2016) or based on the results of echocardiography, cardiac catheterization detection, HE staining of cardiomyocytes, and mRNA levels of related genes (Liu et al., 2018; Zhang et al., 2020).

**Table 4:** ELF-EMF's effects on the cardiovascular system.

**References**	***In vitro/In vivo***	**Intervention description**	**Animal species**	**Main effect/Possible cause/ Mechanism**
Elmas et al. (2012)	*In vivo*	50-Hz, 1-μT EMF	Wistar albino rats	No effect was observed.
Jeong et al. (2005)	*In vivo*	EMF (60 Hz, 20 G) for 1 (MF-1) or 5 days (MF-5)	Male rats	In short-term exposure, the results indicated that ELF-EMF might influence ventricular repolarization, leading to a suppression in heart rate elevation.
Liu et al. (2018)	*In vivo*	30 μT, 100 μT and 500 μT EMF exposure for 24 weeks, 20 h per day	SD rats	The results of echocardiography and cardiac catheterization showed that there were no significant differences in cardiac morphology and hemodynamics between the exposed group and the control group.
Martínez-Sámano et al. (2010)	*In vivo*	ELF-EMF (60 Hz, 2.4 mT) for 2 h	Adult male Wistar rats	The reduction of glutathione content in the heart was also detected in a 2-h exposure.
Wei et al. (2015)	*In vitro*	A 2 mT, 15 Hz, 50 Hz, 75 Hz and 100 Hz EMF	Cardiomyocytes isolated from neonatal SD rats	The increase in the calcium concentration baseline level and the decrease in the amplitude of calcium transients in the sarcoplasmic reticulum.
Zhang et al. (2020)	*In vivo*	500 μT EMF exposure for 24 weeks, 20 h per day	Eight-week-old male Sprague-Dawley rats	The heart rate, blood pressure, and pulse rate were not influenced by EMF exposure. HE staining showed no change in the morphology and arrangement of cardiomyocytes.
Zhou et al. (2016)	*In vivo*	A 50-Hz MF at 100 μT for 24 weeks, 20 h per day	Sprague-Dawley (SD) rats	No obvious effects on the cardiovascular system were detected through the detection of blood pressure, pulse rate, heart rate, and cardiac rhythm.

In recent years, EMF therapy has gained significant interest due to its potential protective impact on cardiovascular functions, and various *in vivo* experiments on humans have been conducted. For instance, a pilot randomized controlled trial on healthy adults showed that a magnetic field of 50 Hz had therapeutic effects to stimulate parasympathetic activity, resulting in enhanced vasodilation and blood flow through a nitric oxide-dependent mechanism (Okano et al., 2021). A double-blind, repeated study explored the effect of ELF-EMFs on heart rate (HR) and HR variability and found a reduction in HR caused by the possible enhancement of parasympathetic predominance by short-term EMF (Binboga et al., 2021). Another study exposed mild-to-moderate hypertension patients to 1 μT ELF-EMF and observed a blood pressure-lowering effect (Nishimura et al., 2011). The phenomenon of EMF-related vasodilation and microcirculation was also detected clinically (Kondo et al., 2019).

Apart from the observed phenomenon, the mechanism behind was further studied. The industry generally believed that despite the lack of a complete understanding of the precise mechanisms, the main mechanisms of action involved the modulation of the autonomic nervous system (ANS), promotion of angiogenesis, enhancement of nitric oxide (NO) synthesis, and exhibition of antioxidant and anti-inflammatory properties (Soltani et al., 2023). Based on that, EMF-related medical devices have been subsequently developed to treat cardiovascular diseases such as hypertension and heart failure non-invasively. Undoubtedly, the emergence of these therapeutic devices has bridged gaps in conventional treatments, yet the substantial benefits of such therapeutic approaches and the specific mechanisms of these modulations still require validation through both *in vivo* and *in vitro* experiments.

### 3.5. Nervous system

Many diseases are concerned with the malfunction of the biological neural system, and thus the association between ELF-EMFs and neural function has attracted some interest. *In vitro* experiments exhibited the potential for cellular behavior modulation by EMF. For instance, immature cerebellar granule neurons (CGNs) of postnatal rats were saved from apoptosis by exposure to the ELF-EMF of 300 mT at 50 Hz, compared with no survival of CGNs without exposure (Oda and Koike, 2004). Ma et al. (2016) found that the proliferation of embryonic neural stem cells (eNSCs) was enhanced under the exposure of 1 mT, 50 Hz ELF-EMF. Furthermore, EMF has the potential to facilitate the differentiation of neural lineages and enhance the maturation of neurons (Ho et al., 2019).

In addition to the cellular-level effects, *in vivo* experiments were conducted to explore the system-level impacts as summarized in Table 5. Dong et al. (2023) described that it is insufficient to directly activate neuronal action potentials by exposing them to ELF-EMF on the order of milliTesla. However, the ELF-EMFs were able to regulate neural excitability contributing to learning and memory by modulating synaptic plasticity. The effect of ELF-EMF on the spatial memory of mice and rats was complex: Under a short period of exposure, the effect could be ineffective or positive, while EMF could have devastating effects with an increase in frequency, intensity, and exposure duration (Abkhezr et al., 2023). An enhancement of hippocampal neurogenesis in adult mice was also detected after exposure to EMF (Ma et al., 2016). The study conducted by Zheng et al. (2021) explored the impact of EMF stimulation patterns on long-term potentiation (LTP) related to learning and memory at synapses, revealing a consistent decrease in LTP across various waveforms that could be possibly induced by the closure of some calcium channels in the membrane. Rezaei-Tavirani et al. (2018) adopted rat models for the analysis of the proteome. The results indicated that cytoskeletal protein expression increased with the increase in EMF intensity and exposure time, which was possibly induced by the impairment in the short memory of the hippocampus under exposure.

**Table 5:** ELF-EMF's effects on the nervous system.

**References**	***In vitro/In vivo***	**Intervention description**	**Experiment object**	**Main effect/Possible cause/ Mechanism**
Bonmassar et al. (2012)	*In vitro*	Sub-millimeter magnetic coils (FEM predicted the intensity as approximately 0.02 T, 1 Hz)	Ganglion cells from rabbit retina	Sub-millimeter magnetic coils were able to activate neuronal tissue.
Cichoń et al. (2017)	*In vivo*	7 mT at 40 Hz for 15 min/day for 4 weeks	Brain stroke patients	Oxidative stress can be modulated by EMF.
Cichoń et al. (2018)	*In vivo*	7 mT at 40 Hz for 15 min/day for 4 weeks	Brain stroke patients	Exposure to ELF-EMF led to a significant elevation in the levels of growth factors and cytokines associated with neuroplasticity and facilitated a notable improvement in functional recovery.
Gao et al. (2021)	*In vivo*	ELF-EMF (50 Hz, 1 mT) for 2 h daily on 28 successive days	Rat	Hippocampal neurogenesis was enhanced by the ELF-EMF with cerebral ischemia, and the effects may probably be generated through the upregulation of the Notch signaling pathway.
Jeong et al. (2022)	*In vivo*	Micro-coil stimulation	Rat vagus nerve	This way of stimulation was an effective alternative to VNS with fewer heart-related side effects.
Lee and Fried (2015)	*In vitro*	0.5-mm diameter coils (250–1000 Hz)	Subthalamic nucleus (STN) neurons from C57BL/6 mouse brain slice	Sub-millimeter magnetic coils were able to suppress the activation of neuronal tissue.
Lee and Fried (2017)	*In vitro*	Micro-coil stimulation	Layer V pyramidal neurons (PNs)	The coils produced asymmetric fields and activated the corresponding focal region.
Ma et al. (2016)	*In vivo and in vitro*	1 mT, 50 Hz ELF-EMF	Adult mice	An enhancement of hippocampal neurogenesis in adult mice was detected, and the proliferation of embryonic neural stem cells (eNSCs) was enhanced.
Moya-Gómez et al. (2023)	*In vivo*	13.5 mT/60 Hz EMF	Ischemia/reperfusion model on Mongolian gerbils (6-month-old males)	ELF-EMF enhanced the neurological assessment and behavioral outcomes, exhibited a positive impact on neuronal viability, and contributed to a reduction in glial reactivity within the hippocampus.
Oda and Koike (2004)	*In vitro*	ELF-EMF of 300 mT at 50 Hz for 5 days	Cerebellar granule neurons (CGNs) of postnatal rats	CGNs were saved from apoptosis.
Park et al. (2013)	*In vivo*	Micro-coil stimulation	Central nervous systems of hamsters	Inferior colliculus neurons in the dorsal cochlear nucleus were activated.
Rezaei-Tavirani et al. (2018)	*In vivo*	50 Hz ELF-EMF at 0.5 and 1 mT for 2 and 4 weeks	Male rat	This study determined that ELF-EMF led to the change in protein expression related to the cytoskeleton in the rat hippocampus that contributes to major processes in brain damage.
Zheng et al. (2021)	*In vivo*	15 Hz/2 mT ELF-EMF	LTP at the Schaffer collateral-CA1 (SC-CA1) synapses in Sprague–Dawley rats	A consistent decrease in LTP was detected across various waveforms which could be possibly induced by the closure of some calcium channels in the membrane.

Under conditions of cerebral ischemia, EMF has been found to promote neural regeneration in the hippocampus to possibly repair brain tissue through animal behavior experiments, which could be induced by the modulation of the Notch signaling pathway (Gao et al., 2021). For stroke, Cichoń et al. (2017) denoted that oxidative stress can be modulated by the ELF-EMF, which significantly advanced the functional and mental status of patients with a field of 7 mT at 40 Hz. In a follow-up study on post-stroke patients, they found that exposure to ELF-EMF led to a significant elevation in the levels of growth factors and cytokines associated with neuroplasticity and facilitated a notable improvement in functional recovery (Cichoń et al., 2018). In animal models of transient stroke, the results indicated that exposure to ELF-EMF enhanced the neurological assessment and behavioral outcomes, exhibited a positive impact on neuronal viability, and contributed to a reduction in glial reactivity within the hippocampus, which confirmed the potential of EMF as a clinical therapy (Moya-Gómez et al., 2023).

EMFs have demonstrated promising applications in the regulation of neural activities so as to provide a new means for the treatment of nervous system diseases. Compared with traditional therapeutic methods of electrical stimulation, EMFs in this field have advantages such as indirect contact with tissues to reduce potential damage, induction of focal neural activity, and achievement of selective activation of certain neuron populations. For instance, transcranial magnetic stimulation (TMS) is widely applied in treating neurological disorders, notably depression and anxiety (Fregni and Pascual-Leone, 2007; Klomjai et al., 2015; Cappon et al., 2022). Within intricate systems such as the retina and visual cortex, EMFs were able to offer a less-invasive approach to modulating neuronal behavior. Bonmassar et al.'s (2012) and Lee and Fried's (2015) studies introduced sub-millimeter magnetic coils to activate or suppress neuronal tissue through *in vitro* experiments, which offered an effective alternative for deeper and sub-cortical targets. Lee and Fried (2017) further utilized the micromagnetic stimulation on layer V pyramidal neurons (PNs) and showed that the coils produced asymmetric fields and activated the corresponding focal region, which was promising for selective stimulation of local neurons. Park et al. (2013) applied this technology in the central nervous systems of hamsters and activated inferior colliculus neurons in the dorsal cochlear nucleus. In vagus nerve stimulation (VNS), Jeong et al. (2022) adopted micro-magnetic stimulation of the rat vagus nerve and found this way of stimulation to be an effective alternative to VNS with fewer heart-related side effects.

Regarding the underlying mechanisms of these effects, the activities of neuronal ion channels were crucial and basic. According to Bertagna et al. (2021), the most frequently observed outcome of EMF exposure appears to be alterations in calcium balance, primarily associated with voltage-gated calcium channels. Under both acute and chronic exposure to ELF-EMF, an elevation of calcium ion concentration was observed intracellularly, which should be the foundation for neural activity modulation. A more intricate network of causal elements could range from interactions at the cell membrane that initiate signaling pathways to the involvement of various mechanisms such as ROS, nitric oxide, growth factors, cryptochromes, and processes related to epigenetic and genetic alterations (Funk and Fähnle, 2021; Dong et al., 2023). This comprehensive interplay within the neural environment underlines the multifaceted nature of the mechanisms that underlie the effects of EMF on the nervous system.

## 4. Discussion

Currently, human lives are surrounded by various kinds of electromagnetic fields from power transmission lines, home appliances, transformers, and mobile phones. People are paying much attention to the potential health problems associated with ELF-EMFs. Most of these studies are cohort studies, and there may be confounding factors such as age, sex, magnetic induction, and frequency. The ICNIRP also denotes that there are no fixed thresholds for possible adverse biological effects due to inadequate consistent scientific evidence. Consequently, by reviewing the existing literature about the effects of ELF-EMFs on systems, this study aimed to present an overview of biological effects related to ELF-EMFs from an *in vivo* perspective.

It is well apprehensible that electromagnetic field intensity plays a significant role in humans on a system level. In terms of the immune system, ELF-EMFs at 1 mT and 4.5 mT increased thymus, spleen, and macrophage phagocytosis indexes, contributing to biological immune function. The decrease of these indexes happened at a higher intensity of 9 mT, which indicated potential damage to immune function. In terms of the influence on fertility, it was found that an electromagnetic field strength of 1.2 mT decreased the delivery rate of pregnant rats and also led to preterm birth, stillbirth, and teratogenesis. No connection has been detected in mammals after exposure to ELF-EMFs. Studies of their offspring also exposed to EMFs have shown that exposure to extremely low-frequency magnetic fields had no harmful effect on their growth or reproduction. On the one hand, EMF intensity lower than 500 μT showed no obvious influence on delivery rate or mass. On the other hand, ELF-EMFs such as 30 μT, 100 μT, and 500 μT also showed no effect on the heart rhythm, cardiac histology, or blood pressure of SD rats. In the nervous system, EMF had positive effects on some neural cells, such as CGNs and eNSCs. The intensity of milliTesla was not able to activate single neurons but was able to regulate some neural activities. Based on this, studies have been carried out to explore the positive and regulable effects of EMF on hippocampus-related synaptic and neural plasticity and on post-stroke rehabilitation. TMS and millimeter magnetic coils were also adopted to treat nervous diseases and modulate the retina and visual cortex.

*In vivo* experiments, investigating the effects of EMF exposure on cancer in animals have produced mixed results, with varying outcomes reported depending on factors such as the EMF parameters, animal species, and cancer models used. Some studies observed no significant association between EMF exposure and cancer incidence (e.g., mammary murine adenocarcinoma in mice and colon cancer in rats), while others detected increased incidence of specific tumors, indicating the complex and context-dependent nature of EMF–cancer interactions (e.g., adenocarcinoma in rats). The potential carcinogenic mechanisms of ELF-EMF involve factors such as DNA damage, impaired cellular repair processes, altered hormone secretion, and oxidative stress. Discrepancies in *in vivo* outcomes about DNA damage could be attributed to genetic variations and cell sensitivity. The hypothesis of EMF's impact on melatonin reduction in the pineal gland was not consistently supported by experimental results. Since ELF-EMFs have demonstrated a potential for inducing apoptosis in tumor cells, EMFs were studied for therapeutic uses. *In vivo* research on mice highlighted a significant increase in proinflammatory responses and inhibition of tumor growth, which was attributed to ROS accumulation and Ca^2+^ overload-induced necroptosis.

From the review of the literature, ELF-EMFs on the order of mT could probably affect the organism cellularly or systematically. Sometimes, the influence is helpful and therapeutic. Accumulating evidence demonstrates that ELF-EMFs are capable of modifying neuronal function with magnetic fields on the order of mT. For low-dose magnetic field exposure, such as those weaker than 500 μT, especially 100 μT or 200 μT, there are no obvious effects on biological tissue, whether *in vitro* or *in vivo*. Gutiérrez-Mercado et al. (2013) noted that biological systems exposed to EMFs of 0.66 mT can produce health effects such as tissue recovery in wounds, blood circulation re-establishment after tissue ischemia, and increasing vascular permeability of circumventricular organs in the brain. Since the ELF-EMFs surrounding our occupational and living environment are only several μT or <0.1 μT (McCurdy et al., 2001), these findings further give us confidence that biological health would not be harmed by ELF-EMFs in the surroundings from power transmission lines, electrical appliances, base stations, mobile phones, etc. with low-dose radiation exposure.

In addition, this review further sheds light on the application of ELF-EMFs to modulate or treat health problems clinically. High-frequency EMF primarily interacts with biological tissues through thermal effects, where energy is absorbed and converted into heat. This can lead to tissue heating and potential thermal damage, which is also the basis for applications such as medical diathermy and microwave ablation. ELF-EMF's interactions with biological tissues are more complex and are often attributed to non-thermal mechanisms. These include the induction of electric currents in tissues due to their conductive nature, which can affect cellular processes or recruit excitable tissues. After conducting literature research, it becomes evident that EMFs have a significant impact on neural functions such as neuronal electrical signaling. Additionally, there is a promising avenue for further exploration into the applications of electromagnetic fields in neural modulation.

In conclusion, ELF-EMF's effects are diverse and have been proposed to influence various physiological and biochemical processes, such as ion channel activities, DNA damage, hormone secretion, and oxidative stress reactions. In order to fully harness the therapeutic potential of EMF, it is essential to conduct comprehensive research into the underlying mechanisms of its effects on biological tissues so as to lay the foundation for precise modulation and control of EMF therapies, enabling their optimal utilization.

## 5. Conclusion

To sum up, the ELF-EMFs of different frequencies and intensities will have various effects on biological activities, either microscopically or macroscopically. Due to inconsistent model establishment and instability in the frequency and exposure time of ELF-EMFs, the research on the health effects of ELF-EMFs cannot come to a consistent conclusion. However, under weak magnetic fields, most of the research supported the fact that there are no obvious adverse effects of ELF-EMFs on biological organisms. In particular, more and more interest is being paid to adopt ELF-EMFs for disease treatment. Consequently, within the daily-life environment, ELF-EMFs would not affect human health, and they can be further applied to treat specific diseases under some medium magnetic fields.

## Author contributions

HT contributed to design, drafting, and revision of this paper. HZ actively contributed to the original drafting of this paper. CG, MS, DY, and MJ contributed to figure drawing and manuscript revision. FW and XS contributed to design and revision of this paper. All authors contributed to the article and approved the submitted version.
